# Exposure to Neighborhood Violence and Gun Carrying Among Adolescents in the United States: Findings From A Population-Based Study

**DOI:** 10.1177/08862605241231616

**Published:** 2024-02-15

**Authors:** Philip Baiden, Yangjin Park, Catherine A. LaBrenz, Saltanat Childress

**Affiliations:** 1 The University of Texas at Arlington, USA

**Keywords:** neighborhood violence, gun carrying, victimization, vulnerability/self-protection, adolescents

## Abstract

Although studies have investigated and found an association between victimization and weapon carrying, few studies have examined the association between exposure to neighborhood violence (NV) and gun carrying among adolescents. The objective of this study was to examine the cross-sectional association between exposure to NV and gun carrying among adolescents. Data for this study came from the 2021 Youth Risk Behavior Survey. An analytic sample of 17,033 adolescents aged 14 to 18 years old (51.7% male) was analyzed using logistic regression with complementary log-log link function. The outcome variable investigated in this study is gun carrying and was measured as a binary variable, whereas the main explanatory variable examined in this study was exposure to NV, which was also measured as a binary variable. Of the 17,033 adolescents, 4.2% carried a weapon during the past year, and 18.7% were exposed to NV. Controlling for the effects of other factors, adolescents who were exposed to NV had more than double the odds of carrying a gun when compared to their counterparts not exposed to NV (adjusted odds ratio = 2.33, 95% Confidence Intervals [1.69, 3.23]). Other significant factors associated with gun carrying include being a male, non-Hispanic Black, being threatened or injured with a weapon, use of alcohol, cigarette smoking, and misuse of prescription opioids. High parental monitoring was protective against gun carrying. The findings of this study underscore the importance of developing age-appropriate intervention strategies to reduce gun carrying among adolescents. School counselors and other professionals working with adolescents in disadvantaged neighborhoods should actively engage parents in assessments and interventions.

## Introduction

Gun carrying among adolescents in the United States (U.S.) is a major public health concern for school personnel, parents, researchers, and policymakers. Between 1999 and 2017, about 39,000 gun-related deaths occurred among school-age children in the U.S. (Rubenstein et al., 2019). Kann et al. (2018) examined data from the National Youth Risk Behavior Survey (YRBS) and found that in 2017, 15.7% of adolescents carried a weapon (e.g., gun, knife, or club) at least once during the past 30 days, whereas 4.8% carried a gun at least once during the past 12 months. Data from the most recent YRBS further indicate that about 4% of adolescents in the U.S. carried a gun in 2021 (Harper et al., 2023). Gun carrying among adolescents can endanger adolescents’ safety and impact learning outcomes (Docherty, Sweeten et al., 2020; Mukherjee et al., 2022). Yet, few studies have focused on examining factors associated with gun carrying among adolescents, drawing on nationally representative samples, partly due to the fact that the prevalence of gun carrying among adolescents is low relative to other weapons (Gunn III & Boxer, 2022; Kann et al., 2018), and gun carrying is often times combined with other weapons such as knives or clubs (Lowry et al., 2023; Pham et al., 2017). It is important to identify factors associated with gun carrying among adolescents. Such identification could help in risk assessment of adolescents who are more likely to carry a gun with important intervention and prevention strategies developed to prevent future gun-related violent incidents.

Available research indicates that adolescents may carry weapons both in and outside school for self-protection due to the fear of being victimized and negative perceptions of their own safety (Semprevivo et al., 2020). Whereas studies have found that factors such as sexual violence (SV) victimization (Baiden et al., 2022; DuPont-Reyes et al., 2014), bullying victimization (Ganson & Nagata, 2021; Perlus et al., 2014; Pontes & Pontes, 2021a), and being threatened with a weapon (Mukherjee et al., 2022; Vaughn et al., 2006) are frequently associated with general weapon carrying among adolescents, one important factor that has received relatively less research attention is the association between exposure to neighborhood violence (NV) and gun carrying among adolescents (Beardslee et al., 2018; Ray, 2022).

Existing studies that have investigated the association between exposure to NV and gun carrying among adolescents are less diverse, either focused on a specific population, such as justice-involved adolescents (Ray, 2022), non-Hispanic Black adolescents (Spano, 2012; Spano et al., 2012), or adolescents from a specific geographical location in the U.S. (Beardslee et al., 2018), which limit the generalizability of the findings. For instance, Spano and Bolland (2011) examined data from a longitudinal sample of racial/ethnic minority adolescents living in extreme poverty from Mobile, Alabama, and found that the effect of exposure to NV at Time 1 on initiation of gun carrying at Time 2 became non-significant after adjusting for other violent and victimization-related behaviors at Time 1. Beardslee et al. (2018) investigated exposure to NV among a sample of 1,170 male juvenile offenders (14–19 years) recruited from Arizona and Pennsylvania and found that adolescent offenders who were exposed to NV were significantly more likely to carry a gun after exposure to gun violence, but not after exposure to non-gun violence.

The literature regarding the association between mental health problems and gun carrying is inconclusive. Whereas there is little evidence to suggest that individuals with mental health problems, especially depression or anxiety, are more likely to perpetrate violent crimes (Senior et al., 2020), higher rates of violence perpetration have been observed among individuals with some serious mental illness, particularly bipolar disorder and schizophrenia (Green et al., 2019). Thus, some scholars have argued that individuals with depression and anxiety are rather more likely to be victims of crime rather than perpetrators of crime (Thornicroft, 2020). The association between substance use and gun carrying among adolescents has been well established, with research showing that adolescents who use alcohol, smoke cigarettes, use marijuana, or illicit drugs are more likely to carry a gun (Baiden et al., 2021; Docherty, Mulvey et al., 2020; Ganson & Nagata, 2021; Pontes & Pontes, 2021b). With respect to demographic factors, the literature is fairly consistent that compared to adolescent females, adolescent males are more likely to carry a gun (Beardslee et al., 2018; Khubchandani & Price, 2018; Ray, 2022). Other studies have also found the prevalence of gun carrying among non-Hispanic Black adolescents is higher than among adolescents of other racial/ethnic identities (Spano, 2012; Spano et al., 2012).

Although most studies have focused on risk factors associated with gun carrying (Beardslee, Docherty et al., 2021; Docherty, Mulvey et al., 2020; Docherty, Sweeten et al., 2020; Pontes & Pontes, 2021b; Ray, 2022; Simon et al., 2022), it is equally important to take into account factors that might be protective against violent behaviors. Instead of focusing solely on identifying adolescents who might be at risk of carrying a gun, studies could also identify protective factors against gun carrying among adolescents. Parental monitoring has been identified as protective against adolescents’ health risk behaviors such as suicide, substance use, and risky sexual behaviors (Booth & Shaw, 2023; Villarreal & Nelson, 2018; Voisin et al., 2012).

### Theoretical Framework

This study is guided by the vulnerability/self-protection theory (Valdebenito et al., 2017). The vulnerability/self-protection theory posits that individuals who have been victimized may turn to weapon carrying as a means of self-protection (Valdebenito et al., 2017; Van Geel et al., 2014). Adolescents exposed to NV may experience a heightened sense of fear and, as a result, may choose to protect themselves from perceived threats in the neighborhood environment. This line of reasoning is further supported by research demonstrating that self-protection is one of the most commonly cited reasons for weapon carrying among adolescents who have been victimized (Baiden et al., 2022; Semprevivo et al., 2020). Systematic reviews and meta-analyses have also found that prior victimization and perception of imminent danger or threat, either real or assumed, may play a direct role in identifying weapon carrying as a form of self-protection (Valdebenito et al., 2017; Van Geel et al., 2014).

### Current Study

Although studies have investigated and found an association between victimization and weapon carrying among adolescents, to our knowledge, few studies have investigated the association between exposure to NV and gun carrying among adolescents, drawing on a large nationally representative sample (Harper et al., 2023; Simon et al., 2022). Most of the existing studies on exposure to NV and gun carrying tend to focus on adults (Carter et al., 2020; Sokol et al., 2022). Thus, drawing on a large and diverse nationally representative sample, the current study seeks to fill the knowledge gap by examining the cross-sectional association between exposure to NV and gun carrying among adolescents. We hypothesized that controlling for demographic, mental health, and substance use factors, exposure to NV, and prior victimization would be significantly positively associated with gun carrying. We further hypothesized that parental monitoring would be associated with lower likelihood of gun carrying.

## Methods

### Data Source and Participants

Data for this study came from the 2021 YRBS. The YRBS is a cross-sectional, school-based national survey conducted by the Centers for Disease Control and Prevention (CDC) every 2 years to ascertain the prevalence, patterns, and co-occurrence of health risk behaviors that contribute to the leading causes of death and disability among adolescents in the U.S. and to monitor progress toward achieving the Healthy People objectives. The YRBS recruited 9th to 12th graders from both public and private schools to complete self-administered surveys. The sample is diverse by the design with respect to race/ethnicity and sexual identity. Detailed information about the YRBS, including the objectives, methodology, and sampling procedure, has been provided elsewhere (Brener et al., 2013; Mpofu et al., 2023).

### Sample

There were 17,232 respondents in the 2021 YRBS; however, the analyses conducted in this study were based on 17,033 adolescents aged 14 to 18 years. The percent of missing data ranged from less than 1% for age to 46% for illicit drug use. Missing data analysis was conducted to assess whether a group of respondents with observed data on one variable is significantly different from a group of respondents with missing data on another variable. We found that data were missing completely at random (MCAR); that is, the probability of missingness on one variable was not dependent on any observed data or unobserved data (Van Ginkel et al., 2020). Given that data were MCAR, Multiple Imputation using Chained Equations was chosen as the most appropriate technique to impute complete data (Van Buuren, 2018). We followed the four steps recommended by Azur et al. (2011) in imputing missing data and generated 20 imputed datasets. This number is generally considered sufficient to improve the model’s robustness (Azur et al., 2011; Graham et al., 2007). A similar approach has been used by the authors in previous studies (Baiden et al., 2019, 2020, 2021, 2022, 2023, 2024; Baiden & Tadeo, 2020).

### Variables

#### Outcome Variable

The outcome variable investigated in this study is gun carrying and was measured as a binary variable. Adolescents were asked, “During the past 12 months, on how many days did you carry a gun? (Do not count the days when you carried a gun only for hunting or for a sport, such as target shooting.)” with the following response options “1 = 0 days,” “2 = 1 day,” “3 = 2 or 3 days,” “4 = 4 or 5 days,” and “5 = 6 or more days.” Due to small cell counts in higher-order categories and for practical utility from an analytic perspective, adolescents who indicated carrying a gun at least once during the past 12 months were recoded as 1. In contrast, adolescents who did not carry a gun during the past 12 months were recoded as 0.

#### Explanatory Variable

The main explanatory variable examined in this study was exposure to NV and was measured as a binary variable based on response to the question, “Have you ever seen someone get physically attacked, beaten, stabbed, or shot in your neighborhood?” Adolescents who answered “yes” were coded as 1, whereas adolescents who answered “no” were coded as 0.

#### Covariates

**Figure 1.**
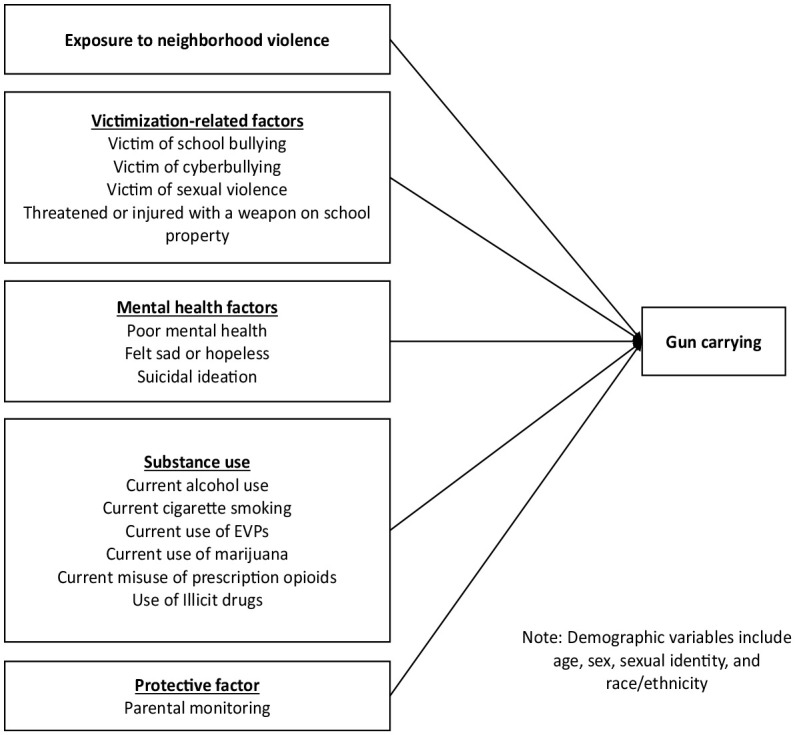
List of variables examined in this study.

As shown in Figure 1, covariates examined were grouped under the following: victimization-related factors (victim of school bullying, cyberbullying, SV, and being threatened or injured with a weapon on school property), mental health factors (poor mental health, feeling sad or hopeless, and suicidal ideation), and substance use (alcohol, cigarette smoking, electronic vaping products (EVPs), marijuana, misuse of prescription opioids, and illicit drugs). The exact wording of the questions and analytic coding for each covariate are provided in Table 1.

**Table 1.** List of Covariates Derived From the 2021 YRBS.

Variable Name	Question	Analytic Coding
Victimization-related factors		
Victim of school bullying	During the past 12 months, have you ever been bullied on school property?	No vs. Yes
Victim of cyberbullying	During the past 12 months, have you ever been electronically bullied? (Count being bullied through texting, Instagram, Facebook, or other social media.)	No vs. Yes
Victim of sexual violence	During the past 12 months, how many times did anyone force you to do sexual things that you did not want to do? (Count such things as kissing, touching, or being physically forced to have sexual intercourse.)	0 times vs. 1 or more times
Threatened or injured with a weapon on school property	During the past 12 months, how many times has someone threatened or injured you with a weapon such as a gun, knife, or club on school property?	0 times vs. 1 or more times
Mental health factors		
Poor mental health	During the past 30 days, how often was your mental health not good? (Poor mental health includes stress, anxiety, and depression.)	Most of the time/Always vs. Sometimes/Rarely/Never
Feeling sad or hopeless	During the past 12 months, did you ever feel so sad or hopeless almost every day for 2 weeks or more in a row that you stopped doing some usual activities?	No vs. Yes
Suicidal ideation	During the past 12 months, did you ever seriously consider attempting suicide?	No vs. Yes
Substance use factors		
Current alcohol use	During the past 30 days, on how many days did you have at least one drink of alcohol?	0 days vs. 1 or more days
Current cigarette smoking	During the past 30 days, on how many days did you smoke cigarettes?	0 days vs. 1 or more days
Current use of EVPs	During the past 30 days, on how many days did you use an electronic vapor product?	0 days vs. 1 or more days
Current use of marijuana	During the past 30 days, how many times did you use marijuana?	0 days vs. 1 or more days
Current misuse of prescription opioids	During the past 30 days, how many times did you take prescription pain medicine without a doctor’s prescription or differently than how a doctor told you to use it?	0 days vs. 1 or more days
Use of illicit drugs	Ever use of one of the following illicit drugs: cocaine, inhalants, heroin, methamphetamines, ecstasy, or hallucinogens, one or more times during their life)	Never vs. at least once
Protective factor(s)		
Parental monitoring	How often do your parents or other adults in your family know where you are going or with whom you will be?	Most of the time/Always vs. Sometimes/Rarely/Never

*Note.* EVPs = electronic vaping products; YRBS = Youth Risk Behavior Survey.

We also took into account parental monitoring as a protective factor against gun carrying. Parental monitoring was measured based on responses to the question, “How often do your parents or other adults in your family know where you are going or with whom you will be?” with the following response options, “never,” “rarely,” “sometimes,” “most of the time,” and “always.” For the purposes of this study and following the recommendation of other scholars (Dittus et al., 2023), adolescents who indicated “never,” “rarely,” or “sometimes” were considered to have low parental monitoring and were coded as 0, whereas adolescents who indicated “most of the time,” or “always” were considered to have high parental monitoring and were coded as 1.

#### Demographic Variables

We controlled for the following demographic variables. Age was measured in years, whereas sex was coded as “0 = Female” versus “1 = Male.” Sexual identity was coded as a nominal variable into “0 = Straight,” “1 = Lesbian/gay,” “2 = Bisexual,” and “3 = Other/questioning.” Race/ethnicity was coded as a nominal variable into the following categories “0 = non-Hispanic White,” “1 = non-Hispanic Black,” “2 = Hispanic,” “3 = Asian,” “4 = Native American/American Indian,” and “5 = Other race/ethnicity.”

### Data Analyses

Data were analyzed using descriptive and multivariable analytic techniques. The general distribution of all the variables included in the analysis was first examined using percentages. Bivariate associations among the explanatory and control variables were examined to check for the presence of multicollinearity. None of the variables had a variance inflation factor greater than four to pose a problem of multicollinearity. The main analysis involves using complementary log-log models to estimate the association between exposure to NV and gun carrying while simultaneously controlling for the effects of risk and protective factors. We opted for complementary log-log function over logistic or probit function given that our outcome variable was heavily skewed, with 4.2% of adolescents reported carrying a gun. The complementary log-log function is most appropriate when the occurrence of the binary outcome is very rare (Agresti & Finlay, 2008). Three regression models were fitted with variables entered in a hierarchical order. In Model 1, we regressed gun carrying on exposure to NV while controlling for demographic factors due to their a priori importance. Model 2 consists of variables in Model 1 plus victimization-related, mental health, and substance use factors. The fully adjusted model consists of variables in Model 2 plus parental monitoring. Adjusted odds ratios (AORs) are reported together with their 95% Confidence Intervals (CI). Variables were considered significant if the *p-*value was less than .05. Stata’s “svyset” command was used to adjust for the weighting and complexity of the multistage cluster sampling design employed by the YRBS. All analyses were performed using STATA 17 MP (Stata Corp., College Station, TX, USA).

## Results

### Sample Characteristics

The distribution of the study variables is presented in Table 2. Of the 17,033 adolescents examined, 4.2% reported carrying a gun during the past 12 months. About one in five adolescents (18.7%) were exposed to NV. About 16.1% of adolescents were victims of school bullying and cyberbullying, 11.5% were victims of SV, and 6.6% were threatened or injured with a weapon on school property. About 4 in 10 adolescents (39.8%) reported feeling sad or hopeless, 28.3% had poor mental health, and 21.3% experienced suicidal ideation during the past 12 months. The distribution of substance use factors is as follows: current alcohol use (23.1%), current use of EVPs (19.2%), current use of marijuana (15.8%), use of illicit drugs (12.5%), current misuse of prescription opioids (5.7%), and current cigarette smoking (4.0%). About seven in eight adolescents (86.4%) had high parental monitoring. The proportion of adolescents exposed to NV and carrying a gun was 12.9%, significantly greater than the proportion of adolescents not exposed to NV and carried a gun (2.9%; OR = 5.98, *p* < .001, 95% CI [4.53, 7.90]). Of the demographic factors examined, the proportion of adolescents carrying a gun was greater for adolescents who are 18 years old, male, or self-identify as non-Hispanic Black. The proportion of adolescents that reported carrying a gun was greater if they were victims of school bullying, cyberbullying, SV, or were threatened with a weapon on school property. At the bivariate level, adolescents were more likely to carry a gun if they had poor mental health, reported feeling sad or hopeless, or had suicidal ideation. All the substance use factors examined were significantly associated with gun carrying at the bivariate level. Also, 3.2% of adolescents with high parental monitoring compared to 14.3% of adolescents with low parental monitoring reported carrying a gun (OR = 0.17, *p* < .001, 95% CI [0.14, 0.22]).

**Table 2.** Sample Characteristics (*n* = 17,033).

Variables	Frequency (Weighted %)	Gun Carrying		OR [95% CI]	*p*-value
		No (%)	Yes (%)		
Outcome variable					
Carried a gun					
No	16,312 (95.8)				
Yes	721 (4.2)				
Explanatory variable					
Exposure to neighborhood violence					
No	13,842 (81.3)	97.1	2.9	Reference category	
Yes	3,191 (18.7)	87.1	12.9	5.98 [4.53–7.90]	<.001
Demographic variables					
Age					
14 years	3,403 (20.0)	95.8	4.2	Reference category	
15 years	4,427 (26.0)	95.6	4.4	1.01 [0.71–1.43]	.956
16 years	4,276 (25.1)	95.0	5.0	1.16 [0.81–1.66]	.425
17 years	3,904 (22.9)	94.9	5.1	1.14 [0.80–1.63]	.459
18 years	1,023 (6.0)	92.6	7.4	2.12 [1.28–3.49]	.004
Sex					
Female	8,224 (48.3)	97.5	2.5	Reference category	
Male	8,809 (51.7)	92.9	7.1	2.83 [2.13–3.76]	<.001
Sexual identity					
Straight	12,762 (74.9)	95.1	4.9	Reference category	
Lesbian/gay	525 (3.1)	93.6	6.4	1.01 [0.44–2.34]	.983
Bisexual	1,906 (11.2)	96.4	3.6	0.75 [0.47–1.20]	.223
Other/questioning	1,840 (10.8)	94.8	5.2	1.39 [0.97–1.98]	.072
Race/ethnicity					
Non-Hispanic White	9,308 (54.6)	96.0	4.0	Reference category	
Non-Hispanic Black	2,353 (13.8)	93.4	6.6	1.71 [1.34–2.20]	<.001
Hispanic	3,265 (19.2)	94.0	6.0	1.24 [0.93–1.66]	.147
Asian	851 (5.0)	97.7	2.3	0.30 [0.11–0.81]	.019
Native American/American Indian	239 (1.4)	87.2	12.8	1.76 [0.76–4.09]	.182
Other race/ethnicity	1,017 (6.0)	95.6	4.4	0.92 [0.45–1.89]	.818
Victimization-related factors					
Victim of school bullying					
No	14,288 (83.9)	95.9	4.1	Reference category	
Yes	2,745 (16.1)	90.0	10.0	2.70 [2.05–3.55]	<.001
Victim of cyberbullying					
No	14,291 (83.9)	96.0	4.0	Reference category	
Yes	2,742 (16.1)	91.5	8.5	2.57 [2.01–3.29]	<.001
Victim of sexual violence					
No	15,078 (88.5)	95.9	4.1	Reference category	
Yes	1,955 (11.5)	91.9	8.1	3.14 [2.30–4.31]	<.001
Threatened or injured with a weapon on school property					
No	15,904 (93.4)	96.6	3.4	Reference category	
Yes	1,129 (6.6)	76.5	23.5	9.05 [6.77–12.11]	<.001
Mental health factors					
Poor mental health					
No	12,219 (71.7)	95.3	4.7	Reference category	
Yes	4,814 (28.3)	95.0	5.0	1.32 [1.03–1.68]	.027
Feeling sad or hopeless					
No	10,252 (60.2)	95.9	4.1	Reference category	
Yes	6,781 (39.8)	94.1	5.9	1.61 [1.31–1.98]	<.001
Suicidal ideation					
No	13,408 (78.7)	95.9	4.1	Reference category	
Yes	3,625 (21.3)	92.5	7.5	2.35 [1.85–2.97]	<.001
Substance use factors					
Current alcohol use					
No	13,106 (76.9)	96.2	3.8	Reference category	
Yes	3,927 (23.1)	72.3	27.7	4.96 [3.67–6.71]	<.001
Current cigarette smoking					
No	16,360 (96.0)	97.3	2.7	Reference category	
Yes	673 (4.0)	88.6	11.4	10.10 [7.22–14.14]	<.001
Current use of EVPs					
No	13,762 (80.8)	96.9	3.1	Reference category	
Yes	3,271 (19.2)	86.6	13.4	5.54 [4.03–7.61]	<.001
Current use of marijuana					
No	14,336 (84.2)	97.2	2.8	Reference category	
Yes	2,697 (15.8)	87.3	12.7	5.82 [4.36–7.77]	<.001
Current misuse of opioids					
No	16,063 (94.3)	96.4	3.6	Reference category	
Yes	970 (5.7)	77.7	22.3	8.29 [5.97–11.52]	<.001
Use of illicit drugs					
No	14,908 (87.5)	96.8	3.2	Reference category	
Yes	2,125 (12.5)	84.0	16.0	6.00 [4.72–7.63]	<.001
Protective factor(s)					
Parental monitoring					
Low	2,311 (13.6)	85.7	14.3	Reference category	
High	14,722 (86.4)	96.8	3.2	0.17 [0.14–0.22]	<.001

*Note.* EVPs = electronic vaping products.

### Bivariate Association Between Demographic Characteristics and Exposure to NV

Table 3 shows the results of the bivariate association between demographic factors and exposure to NV. Older adolescents were more likely to be exposed to NV (*OR* = 1.05, *p* = .044, 95% CI [1.0, 1.10]). Adolescents who self-identified as bisexual had 1.74 times higher odds of being exposed to NV when compared to adolescents who self-identified as straight (*OR* = 1.74, *p* < .001, 95% CI [1.47, 2.05]). Compared to non-Hispanic Whites, the odds of being exposed to NV were 2.40 times higher for non-Hispanic Blacks (*OR* = 2.40, *p* < .001, 95% CI [1.88, 3.06]), 2.03 times higher for Hispanics (*OR* = 2.03, *p* < .001, 95% CI [1.67, 2.47]), and 1.78 times higher for American Indian/Native Hawaiian/Pacific Islander (*OR* = 1.78, *p* = .008, 95% CI [1.18, 2.71]). Adolescents who self-identified as Asians had 39% lower odds of being exposed to NV when compared to their non-Hispanic White counterparts (*OR* = 0.61, *p* = .004, 95% CI [0.43, 0.85]). Sex was not significantly associated with exposure to NV.

**Table 3.** Bivariate Association Between Demographic Factors and Exposure to Neighborhood Violence (*N* = 7,663).

Variables	OR [95% CI]	*p*-value
Age in years	1.05 [1.00–1.10]	.044
Sex (Male)		
Female	1.08 [1.00–1.17]	.055
Sexual identity (Straight)		
Lesbian/gay	1.25 [0.95–1.64]	.109
Bisexual	1.74 [1.47–2.05]	<.001
Other/questioning	1.17 [0.98–1.40]	.084
Race/ethnicity (Non-Hispanic White)		
Non-Hispanic Black	2.40 [1.88–3.06]	<.001
Hispanic	2.03 [1.67–2.47]	<.001
Asian	0.61 [0.43–0.85]	.004
American Indian/Native Hawaiian/Pacific Islander	1.78 [1.18–2.71]	.008
Other	1.87 [1.45–2.42]	<.001

*Note.* Reference category is indicated in parenthesis. OR = odds ratio.

### Multivariable Logistic Regression Results

Table 4 shows the multivariable logistic regression results examining the association between exposure to NV and gun carrying while controlling for demographic, victimization-related, mental health, substance use, and protective factors. Controlling for demographic factors in Model 1, adolescents who were exposed to NV had more than fivefold higher odds of carrying a gun when compared to adolescents who were not exposed to NV (AOR = 5.74, *p* < .001, 95% CI [4.36, 7.57]). This significant association was partially attenuated with the addition of risk factors in Model 2 and protective factors in Model 3. In the fully adjusted model, exposure to NV was associated with 2.33 times higher odds of carrying a gun (AOR = 2.33, *p* < .001, 95% CI [1.69, 3.23]).

**Table 4.** Multivariable Complementary log-log Regression Results Examining the Association Between Exposure to Neighborhood Violence and Gun Carrying (*n* = 17,033).

Variables	Model 1		Model 2		Model 3	
	AOR [95% CI]	*p*-value	AOR [95% CI]	*p*-value	AOR [95% CI]	*p*-value
Exposure to neighborhood violence (No)						
Yes	5.74 [4.36, 7.57]	<.001	2.51 [1.83, 3.43]	<.001	2.34 [1.69, 3.24]	<.001
Demographic variables						
Age in years	1.09 [1.00, 1.20]	.060	0.98 [0.89, 1.08]	.656	0.96 [0.87, 1.06]	.435
Sex (Female)						
Male	3.06 [2.28, 4.12]	<.001	3.97 [2.82, 5.58]	<.001	3.59 [2.53, 5.08]	<.001
Sexual identity (Straight)						
Lesbian/gay	1.12 [0.48, 2.59]	.792	0.69 [0.26, 1.79]	.435	0.65 [0.25, 1.67]	.361
Bisexual	1.05 [0.64, 1.73]	.830	0.58 [0.34, 0.99]	.046	0.58 [0.34, 0.98]	.042
Other/questioning	1.94 [1.30, 2.89]	.002	1.17 [0.75, 1.81]	.486	1.17 [0.76, 1.80]	.468
Race/ethnicity (Non-Hispanic White)						
Non-Hispanic Black	1.23 [0.93, 1.63]	.152	1.80 [1.27, 2.54]	.001	1.65 [1.16, 2.35]	.006
Hispanic	0.98 [0.73, 1.31]	.864	1.14 [0.83, 1.55]	.404	1.09 [0.80, 1.50]	.573
Asian	0.35 [0.13, 0.90]	.031	0.46 [0.16, 1.30]	.138	0.45 [0.16, 1.23]	.116
Native American/American Indian	1.42 [0.61, 3.31]	.408	1.83 [0.74, 4.52]	.184	1.88 [0.74, 4.80]	.181
Other race/ethnicity	0.72 [0.34, 1.56]	.400	0.72 [0.32, 1.62]	.420	0.71 [0.31, 1.59]	.396
Victimization-related factors						
Victim of school bullying (No)						
Yes			1.28 [0.89, 1.83]	.179	1.35 [0.94, 1.93]	.098
Victim of cyberbullying (No)						
Yes			1.11 [0.79, 1.54]	.540	1.09 [0.79, 1.51]	.599
Victim of sexual violence (No)						
Yes			1.22 [0.80, 1.85]	.350	1.16 [0.77, 1.77]	.465
Threatened or injured with a weapon on school property (No)						
Yes			3.09 [2.24, 4.26]	<.001	2.91 [2.13, 4.00]	<.001
Mental health factors						
Poor mental health (No)						
Yes			1.00 [0.70, 1.42]	.978	0.99 [0.70, 1.41]	.961
Felt sad or hopeless (No)						
Yes			0.79 [0.54, 1.16]	.223	0.77 [0.52, 1.14]	.188
Suicidal ideation (No)						
Yes			1.00 [0.72, 1.40]	.984	0.99 [0.72, 1.37]	.965
Substance use						
Current alcohol use (No)						
Yes			1.95 [1.29, 2.95]	.002	1.87 [1.24, 2.82]	.004
Current cigarette smoking (No)						
Yes			1.70 [1.15, 2.52]	.009	1.67 [1.12, 2.48]	.013
Current use of EVPs (No)						
Yes			1.52 [0.92, 2.52]	.099	1.47 [0.90, 2.42]	.122
Current use of marijuana (No)						
Yes			1.58 [1.01, 2.48]	.046	1.52 [0.98, 2.35]	.064
Current misuse of prescription opioids (No)						
Yes			3.24 [2.11, 4.97]	<.001	3.20 [2.08, 4.91]	<.001
Use of Illicit drugs (No)						
Yes			1.54 [1.11, 2.15]	.012	1.38 [0.99, 1.93]	.060
Protective factor(s)						
Parental monitoring (Low)						
High					0.46 [0.34, 0.62]	<.001

*Note.* Reference category is indicated in parenthesis. AORs = Adjusted odds ratios; EVPs = electronic vaping products.

In the fully adjusted model, the odds of gun carrying were 3.60 times higher for adolescent males when compared to adolescent females (AOR = 3.60, *p* < .001, 95% CI [2.54, 5.10]). Compared to adolescents who self-identified as non-Hispanic White, adolescents who self-identified as non-Hispanic Black had 1.64 times higher odds of carrying a gun (AOR = 1.64, *p* = .007, 95% CI [1.15, 2.34]). We found that controlling for other factors in Model 3, being threatened or injured with a weapon on school property, was associated with 2.92 times higher odds of carrying a gun (AOR = 2.92, *p* < .001, 95% CI [2.13, 4.00]). Other factors associated with gun carrying in Model 3 included current drinking of alcohol (AOR = 1.88, *p* = .004, 95% CI [1.24, 2.84]), current cigarette smoking (AOR = 1.66, *p* = .014, 95% CI [1.12, 2.48]), and misused prescription opioids (AOR = 3.19, *p* < .001, 95% CI [2.08, 4.88]). Controlling for other factors in Model 3, we found that high parental monitoring was significantly associated with 54% lower odds of carrying a gun. (AOR = 0.46, *p* < .001, 95% CI [0.34, 0.62]).

## Discussion

Drawing on a large nationally representative sample, the objective of this study was to examine the cross-sectional association between exposure to NV gun carrying among adolescents. We found that 4.2% of adolescents carried a gun during the past year. This proportion is slightly lower than what some past research that focused on at-risk samples have found (Beardslee, Kan et al., 2021), but at the same time fairly consistent with studies drawing on national data (Ganson & Nagata, 2021; Harper et al., 2023; Simon et al., 2022). The proportion of adolescents exposed to NV (18.7%) fell slightly below that reported in previous research focusing on at-risk populations (Gaylord-Harden et al., 2020).

We hypothesized that exposure to NV and prior victimization would be significantly positively associated with gun carrying while controlling for demographic, mental health, and substance use factors. The findings of this study indicate that exposure to NV and being threatened or injured with a weapon on school property were significantly positively associated with gun carrying among adolescents over and above demographic characteristics, victimization-related factors, mental health, substance use, and protective factors. The findings of this study partly support the notion that gun carrying can be perceived as a response to prior exposure to NV and previous threats. The evidence for this inference is drawn from our findings indicating that both exposure to NV and being threatened or injured with a weapon on school property were significantly positively associated with gun carrying, both having the largest AORs after sex and current misuse of prescription opioids. As the vulnerability/self-protection theory (Valdebenito et al., 2017; Van Geel et al., 2014) postulates, it is possible that our findings that adolescents exposed to NV or threatened or injured with a weapon on school property were more likely to carry a gun could reflect their perception of gun carrying as a means of self-protection. Thus, it is possible that the purpose of adolescents carrying a gun may be a reaction of fear of being re-victimized or re-exposed to NV. Also, the “fear and loathing” hypothesis (Wright et al., 1983) explains that adolescents carrying a gun may fear being targeted for crimes, which may be more prevalent in unsafe neighborhoods. Considering adolescents constantly being exposed to scenes where someone is being physically attacked, beaten, and/or shot at in their neighborhood, adolescents may become more agile and sensitive to any small clues of crime or violence that may increase their likelihood of carrying a gun, without the intention to harm others. Other scholars have also observed that some adolescents who have been victimized, although they might feel an increased need for protection, might at the same time be hesitant in relying on law enforcement for protection due to unpleasant past encounters with law enforcement (Oliphant et al., 2019). Such feelings of hesitation in relying on law enforcement for protection might make some adolescents resort to carrying a gun for self-protection. Moreover, the major uptick in firearm violence during the COVID-19 pandemic and racially motivated traumatic events, such as the George Floyd incident in the summer of 2020 (Howard et al., 2023), may influence our understanding of exposure to NV and gun carrying among adolescents in 2021.

Moreover, it is also possible that association or affiliation with gang membership may contribute to gun carrying. Research has consistently found that gang members are more likely to be involved especially in serious crimes such as gun violence than their non-gang counterparts (Lizotte et al., 2000; Spano & Bolland, 2011; Watkins et al., 2008). Watkins et al. (2008) investigated patterns of gun acquisition, carrying, and use among juvenile and adult arrestees from the city of St. Louis, MO, and found that gang membership was the strongest predictor of gun acquisition, carrying, and use. They found that juvenile arrestees who reported gang membership were five times as likely to report lifetime gun ownership and more than four times as likely to report firing a gun in the past year than non-gang members. Other studies have found gang membership to be associated with exposure to violence either directly as a victim of violent crime for violating gang rules (Peterson et al., 2004) and/or indirectly by witnessing violent crime committed by other gang members or retaliation from rival gang members (Spano et al., 2008; Taylor et al., 2008).

Regarding race/ethnicity, our findings showed that non-Hispanic Black adolescents stood out as a vulnerable population to gun carrying. Prior research has documented that a high rate of gun-related injury and death occurs among non-Hispanic Black adolescents in underprivileged neighborhoods (Spano et al., 2012). Our findings are consistent in identifying non-Hispanic Black adolescents as a salient racial/ethnic group with the elevated risk of exposure to NV and gun carrying compared to their non-Hispanic White counterparts. Among racial/ethnic minority groups, non-Hispanic Black adolescents may be the most underprivileged group that needs special attention and systematic support to reduce the risk of gun carrying. Khubchandani and Price (2018) investigated gun carrying among adolescents between 2001 and 2015 and found although general weapon carrying among school-going adolescents has significantly declined, firearm homicides increased among non-Hispanic Black adolescents over the same period. Furthermore, coupled with the finding that exposure to NV increases the likelihood of gun carrying, this may reflect ongoing structural disparities and racism present in minority neighborhoods that may result in threats to adolescents’ safety without adequate support from police or law enforcement (Bailey et al., 2017), which in turn may increase non-Hispanic Black adolescents’ decision to carry a gun for self-protection.

At the bivariate level, we found that adolescents were more likely to carry a gun if they had poor mental health, reported feeling sad or hopeless, or had suicidal ideation. However, these significant associations disappeared once we controlled for the effects of exposure to NV, demographic, victimization-related, and substance use factors. The non-significant association between mental health factors and gun carrying after adjusting for other factors may suggest that the presence of mental health per se may not necessarily increase the risk of gun carrying among adolescents. And that a constellation of factors might be at play rather than the mere presence of having a mental health problem. Thus, for us to understand the link between mental health and gun carrying among adolescents, it is important to also take into account other important factors such as exposure to NV, history of trauma, and substance use-related factors.

Regarding substance use, we found that alcohol, cigarette smoking, and misuse of opioids were significant factors associated with gun carrying. These findings are consistent with past research that investigated and found adolescent alcohol use and cigarette smoking to be associated with gun carrying (Baiden et al., 2021; Dong, 2021; Ganson & Nagata, 2021). More recently, Pontes and Pontes (2021b) found that misuse of opioids was a critical precursor of gun carrying among adolescents. Some scholars have argued that psychopharmacological effects such as impulsivity and impaired judgment arising from substance use may lead to gun carrying (Chen & Wu, 2016). Others maintained that individuals who use substances carry guns and engage in violent crimes to secure money to further their substance use behaviors (Banks et al., 2017). Thus, substance use and gun carrying among adolescents co-occur and synergistically contribute to more problem behaviors (Dong, 2021). Other studies have also found a robust association between drug dealing and the carrying and use of guns among adolescents (Emmert et al., 2018; Vaughn et al., 2016). Docherty et al. (2019) followed 970 adolescent males ages 14 to 18 from the Pittsburg youth Study and found that for each additional year an adolescent dealt drugs, their odds of carrying a gun increased tenfold for both White and Black adolescents, with stronger association was for Black adolescents. Furthermore, Lizotte et al. (2000) noted that adolescents who sell drugs are more likely to carry guns, possibly defend their territory from competitors, protect their supply from theft, and/or settle violent disagreements. Viewed this way, guns have been described as a “tool of the trade” by adolescent males dealing drugs (Docherty et al., 2020). Due to data limitation, we were unable to assess the link between selling drugs and gun carrying as there was no measure of drug selling in the 2021 YRBS. This is an important area for future research consideration.

In the fully adjusted model, we found that parental monitoring was significantly associated with lower odds of carrying a gun. Although adolescents may be in the process of gradually becoming independent from their parents during the period of adolescence, parental monitoring was a prominent factor in reducing adolescents’ risk of carrying a gun. Consistent with prior research (Booth & Shaw, 2023; Moon et al., 2020; Villarreal & Nelson, 2018; Voisin et al., 2012), parents may still hold a key to protecting adolescents from risky behaviors, including gun carrying. Although gun ownership among adults in the U.S. has increased over the past decades (Stone et al., 2022), the findings of this study indicate that parents who properly monitor their adolescent child can help reduce the risk of gun carrying outside the home. Parents are important figures who may shape adolescent child’s worldview, particularly when it comes to the risk of carrying a gun.

### Study Implications

Findings from this study have important implications for professionals who work with adolescents. First, approximately one in five adolescents in the sample reported exposure to NV. This increased the risk of gun carrying among our sample and has been linked to other negative outcomes for adolescents, including physical health (Jackson et al., 2019) and mental health problems (Meza et al., 2023). Considering that sampling for adolescents in this study occurred in schools, school counselors and administration could consider implementing trauma-informed policies and practices to address exposure to NV (Griffith et al., 2008; Harden et al., 2015). For example, frameworks such as the Truth N’Trauma project have combined a trauma-informed approach with restorative justice to address community violence in schools (Harden et al., 2015). In addition, providing a trauma-informed approach can help support teachers and staff, equipping them to better address responses to adversity, such as NV exposure (Luthar & Mendes, 2020). Second, parental monitoring and involvement served as a protective factor against gun carrying. Some prior research has found that school-based interventions and programs can be an effective way to provide parental education to increase parental monitoring of adolescents (Stormshak et al., 2019) and help prevent violence and victimization (Maas et al., 2022). Thus, school counselors and other professionals working with adolescents in disadvantaged neighborhoods should actively engage parents in assessments and interventions. With regard to policy implications, our findings highlight the importance of critically assessing structural racism and discrimination that may be perpetuated through policies that maintain stratification and inequitable access to resources in certain neighborhoods. Exposure to NV increased the likelihood of gun carrying among adolescents, and there were also racial/ethnic disparities in gun carrying as well as exposure to NV. Prior research has found that racialized adolescents, in particular non-Hispanic Black adolescents, have increased risk of exposure to NV (Browning et al., 2017; Kravitz-Wirtz et al., 2022). Thus, it is important to consider contextual and structural factors that may lead to racial/ethnic disparities and identify structural changes that could be made to increase equity. Finally, while the YRBS is a national sample and reflects diverse groups across race/ethnicity, socioeconomic status, and sexual orientation, it does not necessarily capture youth who are not enrolled in public or private schools (e.g., those who are homeschooled). Moreover, as it is based in the U.S., findings are not necessarily generalizable to youth in other countries.

### Limitations and Direction for Future Research

This study has some limitations that are worth noting. First, this study used cross-sectional data. Hence, causal inferences among the variables cannot be made. For instance, we were unable to determine whether it was the same individual exposed to NV that carried a gun or it was the gun carrying that contributed to exposure to NV. This is an important point for future research consideration. Future research that employs longitudinal design might help us better understand the temporal order between exposure to NV and gun carrying and the potential causal mechanisms involved in this association. Second, the use of secondary data limits our ability to control for other theoretically relevant factors that have been found to be associated with gun carrying, such as gang affiliation or membership, drug dealing, and neighborhood disadvantage. Moreover, despite prior findings on risk factors such as mental health diagnosis (e.g., bipolar disorder, schizophrenia, and psychosis), we were only able to include the symptoms of mental health as actual diagnosis of mental health problems such as bipolar disorder, schizophrenia, and psychosis were not available in YRBS. Future studies should take into account these theoretically important factors to understand the true association between exposure to NV and gun carrying among adolescents. Third, the use of a single item to operationalize some of the measures, such as exposure to NV may affect our findings. Future research using validated scales to capture more nuanced measures is needed. Fourth, while the YRBS is a national sample and reflects diverse groups across race/ethnicity, socioeconomic status, and sexual orientation, it does not necessarily capture adolescents who are not enrolled in public or private schools (e.g., those who are homeschooled). Moreover, as this is a U.S.-based sample, findings are not necessarily generalizable to adolescents in other countries. Since this study was based on adolescents of a school-based sample, out-of-school adolescents (e.g., homeschooling or dropout) were not included in the study. Finally, due to social desirability, it is possible that some adolescent respondents may not report their experiences of gun carrying due to fear of being disciplined.

## Conclusion

In conclusion, drawing on a large nationally representative sample of adolescents, the present study's findings demonstrate an association between exposure to NV and gun carrying over and above other well-established factors known to be associated with gun carrying. School counselors working with adolescents should be aware of some of these factors found to be associated with gun carrying among adolescents. Also, adolescents who were threatened or injured with a weapon in the past or engage in substance use may be a critical target group to reduce the risk of school-based violence, including gun carrying.
